# Defining ‘psychedelic’

**DOI:** 10.1177/02698811261464986

**Published:** 2026-08-17

**Authors:** A. E. Ostrand, M. M. Nour, C. Timmermann, A. Rosati, L. X. Luan, A. Gomez-Emilsson, J. Bornemann, K. T. Greenway, L. Roseman, Carhart-Harris R

**Affiliations:** 1 Carhart-Harris Lab, Department of Neurology, University of California San Francisco, USA; 2 Department of Psychiatry, University of Oxford, UK; 3 Max Planck UCL Centre for Computational Psychiatry and Ageing Research, London, UK; 4 UCL Centre for Consciousness Research, Department of Experimental Psychology, University College London, UK; 5 Centre for Psychedelic Research, Department of Brain Sciences, Imperial College London, UK; 6 Department of Psychiatry and Behavioral Sciences, University of California San Francisco, USA; 7 Department of Psychology, University of Exeter, UK; 8 Qualia Research Institute, San Francisco, CA, USA; 9 Lady Davis Institute, Jewish General Hospital, Montréal, QC, Canada; 10 Department of Psychiatry, Faculty of Medicine, McGill University, Montréal, QC, Canada; 11 Centre for Psychedelic Research, Division of Psychiatry, Department of Brain Sciences, Imperial College London, UK

**Keywords:** psychedelic, dissociative, ketamine, MDMA, empathogen, entactogen, serotonin, NMDA

## Abstract

Humphry Osmond coined the term ‘psychedelic’ in 1956, conjoining ‘psyche’ for ‘soul’ and ‘delic’ for ‘to manifest’ or ‘illuminate’. *Soul-illumination* is a compound noun that describes a psychological state or process. Osmond intended for it to be a taxonomic noun—naming, not just a state—but a category of drug that can induce this psychological effect as its principal action. Consistent with the etymology of *psychedelic*, the present work respects the fundamental importance of phenomenology. Accordingly, we examine the main subjective effect of three different psychoactive drugs, *psilocybin*, *ketamine*, and *MDMA (3,4-methylenedioxymethamphetamine)* (variable label, *Drug*). Over 200 participants rated Delphi-generated subjective rating scale items based on their personal experiences with all 3 drugs. Factor analyses revealed three or four sufficiently independent dimensions of subjective experience (variable label, *Effects*). A machine-learning classifier successfully predicted *Drug* from *Effects*, validating the hypothesis that *psilocybin*, *ketamine* and *MDMA* have categorically distinct subjective effect profiles, differentiable by (1) *visions* and *psychological insight* (*psilocybin*), (2) *dissociation* (*ketamine*) and (3) *pro-social* and *loving feelings* (*MDMA*). We conclude that *psilocybin* is an exemplar *psychedelic*—a category of drug definable by the induction of a *psychedelic state*. The quintessential psychedelic phenomenon is a subjective state characterized by *visions* and *psychological insight*.

## Introduction

One should carve each thing where the natural joint is. Plato. ~427—347 BC.

The term ‘psychedelic’ was devised by British Psychiatrist Humphry Osmond. It was first used, with this spelling, in a letter to Aldous Huxley, written in early April 1956, that opened:

To fathom hell or go angelic

Just take a pinch of PSYCHEDELIC,

(dêlos, to manifest). (Bisbee et al., 2018)

Osmond, like Huxley, was dissatisfied with pre-existing terms for psychoactive drugs such as mescaline and LSD (lysergic acid diethylamide). For example, Osmond called it ‘absurd’ to generically label these compounds ‘psychotomimetic’ (mimicking psychosis) as this is ‘only a small part’ of their range of effects (Osmond, 1957). Three years prior, Osmond favored ‘hallucinogen’ but dropped this too, for a similar reason. Psychoactive drugs, such as LSD and psilocybin, robustly and reliably induce closed-eye visions and other well-characterized visual perceptual effects (Carhart-Harris et al., 2016), but rarely frank hallucinations, and Osmond recognized this. It has also been claimed that ‘hallucinogen’ is an intentionally pejorative term, a hangover of Drug War propaganda, designed to evoke parallels with hallucinations in psychosis (Nutt et al., 2025).

Osmond also considered ‘psychoplastics’—invoking parallels with a recent suggestion, ‘psychoplastogen’ (Olson, 2018). By etymology, *psychoplastogen*—means *making the soul more amenable to change*. At face value, this term has merit, were it not for the fact that it was proposed in association with an altogether very different drug-induced property—post-acute *synaptogenesis*, shown (mostly) in mouse brain. Osmond dismissed ‘psycholytic’ as he felt it connoted ‘dissolution’—which, while an important part of phenomenology of the LSD (and related drug) experience, fails to clearly refer to the illumination of latent psychological content. Osmond felt that this illumination of mental content was the definitive effect of the relevant category of drugs.

Seventy years since the introduction of the term, there remains a remarkable lack of consensus in the scientific community regarding how to define ‘psychedelic’. In the domain of psychometrics, it has been said that there are no ground truths. Be that as it may, it has become customary to refer to an increasingly vague category of compounds as ‘psychedelic’ without any logical, empirical or etymologically sound justification for deciding what can and cannot qualify as a psychedelic. In the present work, we have attempted to advance this matter by using the etymology of ‘psychedelic’ as a guide, acknowledging that it refers to a particular type of *subjective experience* characterized by the manifestation or illumination of content of the mind or psyche that must (by implication) ordinarily be dormant or latent (Bisbee et al., 2018).

*Psychedelic* is a two-component compound neologism (a ‘new word’) that begins with the ancient Greek term ‘psyche’—a noun that originally meant ‘breath’ but became more commonly associated with ‘soul’ and eventually ‘mind’, in more modern times. The term *psychedelic* ends with a morphic adaptation of the ancient Greek adjective ‘dêlos’—which means—making something more ‘visible’, ‘clear’, or ‘manifest’, as in *illuminating* something.

Osmond intended for ‘psychedelic’ to refer to a category of psychoactive drugs. When used in this way, it is both a ‘taxonomic noun’ (it names a thing) and a ‘nominalized adjective’ or ‘descriptive modifier’—that is, it uses a describing word to name a thing, as in ‘a psychedelic x,’ and it can also modify how we perceive a thing, as in ‘x is psychedelic’. Thus, *psychedelic* refers to a property of a subjective state that is characterized by the illumination or manifestation of the latent aspects of the mind. Accepting this, one implication is that other inducers (other than drugs) could cause the *psychedelic state*.

One recent ‘consensus statement’ argued that psychedelic drugs should be defined based on their receptor-level pharmacology, and specifically, agonism of the serotonin 2A receptor (5-HT2AR; Nichols, 2016; Nichols et al., 2023). Another set of authors suggests noun phrases leading with a pharmacological prefix, followed by ‘psychedelic’ as the suffix noun. Some examples include ‘serotonergic psychedelic’ (e.g., psilocybin/psilocin, LSD, DMT, mescaline), ‘glutamatergic psychedelic’ (e.g., ketamine, PCP), ‘GABAergic psychedelic’ (e.g., muscimol; Nutt et al., 2025). This proposal has some appeal. However, if a given compound, such as muscimol, fails to robustly and reliably induce the quintessential psychedelic state, then should we not question categorizing it as a ‘psychedelic’? For example, guided by its phenomenology, might it be better to call it a ‘deliriant’.

There are clear merits to objective (biological) definitions. They circumvent semantic interpretation and cultural biases, for example. But in the current case, effectively defining a category of drug (i.e., psychedelics) by an upstream proteomic action (5-HT2AR agonism) feels vulnerable to category mistakes. The proteomic action (5-HT2AR agonism), is not even a candidate biomarker or correlate for the target phenomenology. Indeed, as we increasingly know, it is likely that there is a specific 5-HT2AR signaling cascade (i.e., not just generic serotonin 2A agonism but a specific form of it) that ultimately leads to a neurophysiology that encodes for the target phenomenology .

Here, we take the position that the quest to identify sensitive and specific biomarkers for the target phenomenology should be maintained, but 5-HT2AR agonism may be a problematic point in the process to place emphasis, as it is far upstream of the key, definitive phenomenology that follows. Constructively, we advocate for an integrative, psychobiological approach, whereby taxonomical decisions can be informed by *both* phenomenological *and* biological assays—that ideally cross-validate each other, that is, they satisfactorily bridge domains because they have a degree of functional equivalence or isomorphism. Relatedly, we advocate for a greater consideration of the original meaning of ‘psychedelic’—that it principally refers to a *quality of subjective experience*, a *subjective state*, in which ordinarily dormant mental phenomena can become manifest. An important question to ask here is: what is its encoding biology?

In this context, Alfred Korzybski’s pithy phrase that ‘the map is not the territory’ is relevant. The aim is to find a taxonomic scheme that does not fall foul of category errors—stemming from a privileging of the map (e.g., 5-HT2AR agonism) over the territory it is intended to point to (psychedelic phenomenology). An example of a *false positive* category error would be to misclassify a drug as ‘psychedelic’ based on its receptor pharmacology (e.g., *Lisuride*, a 5-HT2AR agonist) despite it failing to robustly and reliably induce *psychedelic* subjective effects. An example of a *false negative* would be *ibogaine*, discounted from the family of *psychedelics* because it does not have a well-circumscribed 5-HT2A receptor agonist pharmacology—yet is (arguably) capable of inducing signature *psychedelic* subjective effects. If we were to proceed with a noun phrase approach for ibogaine (Nutt et al., 2025), it would be difficult to select just one prefix, as its pharmacology is complex. The term ‘atypical psychedelic’ could be—and has been—used for compounds such as ibogaine and 5-MeO-DMT. This seems like a reasonable compromise—but only if the suffix correctly applies, for example, a given drug truly has psychedelic subjective effects. Any other policy than this risks creating an inherently vague category.

It is unclear what compounds Osmond wished to include within a category list of bona fide *psychedelics* and which drugs he would exclude. That said, given the focus of his research and the period of history in question, we can confidently regard mescaline and LSD as ‘prototypical psychedelics’. And by this reasoning, the 5-HT2AR agonist definition of psychedelics does classify most prototypical psychedelics, such as LSD, psilocybin/psilocin, dimethyltryptamine (DMT), and mescaline, compounds that others have popularly referred to as the ‘classic psychedelics’ (Nichols, 2016).

However, electing to define psychedelic drugs based on pharmacology alone risks putting the cart before the horse. The most typical and logically sound approach entails putting *phenomenology first* (Frank et al., 2024). Indeed, in the case of *psychedelic drugs*—this is what the *nominalizing adjective* is referring to. Once this principle is recognized, we can work backward to identify the relevant biology that *sensitively* and *selectively* maps to the fundamental, defining subjective experience. This is the integrative ‘psychobiological’ solution that we propose here. The present work aims to begin a process of construct validation to resolve the categorization scheme debate for psychedelics, beginning with a focus on phenomenology.

Psychedelics have re-emerged as a subject matter of considerable scientific and medical interest (Solmi et al., 2022)—and yet a clear and compelling definition—operational or otherwise—for what constitutes a psychedelic remains lacking. Here we aim to improve this situation by introducing and validating a brief operational tool that can successfully parse between the subjective effects of psilocybin or psilocybin mushrooms, and those of two other drugs—that are (arguably) often misclassified as ‘psychedelic’. Specifically, we examine the subjective effects of MDMA and ketamine. MDMA and ketamine were chosen as comparator compounds due to (1) their prevalence of use, (2) their status as a phase 3 experimental medicine and licensed medicine, respectively, and (3) to test the validity of the popular convention of categorizing these compounds as ‘psychedelic’.

A mixed-methods design was employed that involved an initial Delphi process (where experienced, expert opinions are consulted) for deriving subjective rating scale items felt—and found—to be *sensitive* and *specific* to each drug (Anthony, 2025). This was followed by the main phase of this study: an online questionnaire, completed (in full) by an opportunistic sample of over 200 individuals reporting prior personal experience with each of the 3 focal drugs. Statistical analyses were applied to the acquired data, including a machine-learning classifier to test the primary hypothesis that subjective *Effects* could (blindly) predict the specific *Drug* being rated. For brevity and focus, the Delphi component of this study can be found within a Supplemental Material and only the main survey results are presented.

## Methods

### Delphi process

A 3-phase Delphi process was used to derive 18 novel statements that reflect common, subjective effects that the Delphi members regarded—and quantitatively rated—as being especially specific or exclusive to 3 specific psychoactive compounds, namely, psilocybin or psilocybin mushrooms, MDMA, and ketamine. Details of the methods and results of this process can be found within a Supplemental Material.

### Questionnaire process

An online survey component of the study was approved by a local (UCSF) institutional review board and was hosted on Qualtrics^©^ which enables the collection of de-identified survey responses. Key eligibility criteria were 18 years of age or older, at least three prior experiences with each of psilocybin, MDMA and ketamine. Recruitment was conducted via social media posts. The survey asked participants to consider and select a typical dose for each compound/drug (1), as well as a dose that they had taken that had produced *the most intense experience* that they’ve ever had with each compound (2).

Using a visual analog scale format (0–100, with increments of one, where 0 = ‘Not at all’ and 100 = ‘Extreme or Extremely’) participants were asked to rate each of the 18-items derived from the above-described Delphi process based on (1) how well each describes a recent (within the last 6 months) ‘typical experience’ with each drug, as well (2), how well each describes the ‘most intense’ experience with each drug. This generated a total of 36 ratings per participant. Only complete data sets were included in the analysis. Thus, each online survey participant who completed the entire survey gave 2 ratings per item (of which there were 18) for each of the 3 drugs, amounting to ((18 × 3) = 54) × 2 = 108 ratings per (completing) participant. One thousand two hundred and fifty-four responses were collected, of which there were 206 fully complete data sets.

### Statistical analysis

#### Exploratory factor analysis

The factor structure of the 18-item *PKM (Psilocybin Ketamine MDMA)* scale was examined using exploratory factor analysis (EFA) to test our hypothesis that the scale captures distinct dimensions of subjective experience alterations, corresponding to psilocybin, ketamine, and MDMA experiences. Complete datasets from 1236 drug-specific responses were included in the analysis (*n* = 206 participants).

Prior to the EFA, we confirmed that our data were suitable for this factor analysis (Bartlett’s test of sphericity confirmed significant inter-item correlations [χ^2^ = 21058.6, *p* < 10^−10^]; Kaiser-Meyer-Olkin (KMO) measure of sampling adequacy indicated excellent properties [0.89])

We determined the number of factors in two ways. First, we were guided by our prior hypothesis that the data were governed by three factors—informed by prior literature and the design of the Delphi process. A three-factor structure would indicate that the three drugs induce a distinct phenomenology. Second, we used a data-driven approach wherein the questionnaire responses were first subjected to a principal component analysis to determine an inflection point (plateau) in the factor number × eigenvalue plot (Scree plot)—that is, Cattell’s criterion. The factor number corresponding to this inflection point indicates the low-dimensional solution that balances explained variance against model parsimony. This latter approach suggested a four-factor solution.

Thus, we conducted both a three-factor and four-factor EFA. In each case, we used a promax rotation, which allows for correlation between factors rather than assuming orthogonality. This approach was chosen because we did not assume that experiences across the three drug categories would be entirely independent of each other. For each EFA, we examined factor loadings (how individual items mapped onto the extracted factors) and factor scores (how individual drug reports are embedded in a low-dimensional factor space). All statistical analyses were performed in Python (3.10.10, Python Software Foundation) using the scikit-learn and factor-analyzer libraries.

#### Discriminability analysis: Multiclass classification

To quantitatively assess how effectively our EFA solutions discriminated between psilocybin, ketamine, and MDMA experiences, we employed two complementary approaches: a supervised classification model and an analysis of drug centroid separations in factor space.

In multiclass classification, we asked how well drug identity (*psilocybin*, *ketamine*, *MDMA*) could be predicted from its factor representation (i.e., a 3D vector of factor scores). We used a *k*-nearest neighbors algorithm (*k* = 5) for its non-parametric properties and ability to capture non-linear decision boundaries. To ensure unbiased performance estimates, we employed 10-fold cross-validation, stratified by drug type to maintain class proportions across folds. Classification performance was evaluated using balanced accuracy (mean over 10 cross-validation folds) and confusion matrixes.

In the centroid analysis, we calculated the center of mass (centroid) for each drug category in factor space. Drug centroids were computed as the mean factor score vector across all experiences with that drug. We then quantified ‘centroid separation’ as the mean Euclidean distance between every drug-drug pair (psilocybin-MDMA, psilocybin-ketamine, etc.). This can be thought of as a geometric measure of how well the factor solution separates each drug class, complementing the classification approach above.

For both classification and centroid separation analysis, we compared the observed result (prediction accuracy, centroid separation) to a null model generated by 1000 drug label shuffles. A result was deemed significant if its observed metric (prediction accuracy, centroid separation) exceeded 97.5% of the metrics observed under the permutation null distribution (i.e., a two-sided hypothesis test).

#### Dose dependence

To examine whether factor scores are *sensitive* not only to drug identity but also to reported dose, we repeated the above analysis (classification and centroid separation) for subsets of drug experiences, stratifying by reported dosage. In the questionnaire, participants were asked to report the dose corresponding to each drug experience, from a pre-set list (Supplemental Table S1). As these dosages are not directly comparable between drugs, we took a pragmatic approach: for each drug, we sorted the drug experiences according to ascending dose levels, and then quantified the dose range that captured the 0th–25th, 25th–50th, 50th–75th, and 75th–100th percentiles of the sample (e.g., for *MDMA*, first quartile corresponding to the following dose range: 0–50, 51–75, 76–100 mg). This allowed us to quantify classification accuracy and centroid separation as a function of dosage quartile.

## Results

### Delphi process

Results from each iterative phase of the Delphi can be seen in Supplemental Figure S1(a)–(d). The Delphi process generated the final list of 18-items for the PKM scale, shown in Table 1.

**Table 1.** Final items of 18-item PKM scale with average sensitivity and specificity ratings shown.

	PKM scale items	Specificity	Sensitivity
MDMA factor	It was easy to express care and affection.	2.71	1.86
	I felt a strong impulse to connect with those around me.	2.57	2.29
	I experienced a pleasant, reassuring vibe.	2.29	1.57
	I felt an increased wish to be physically close to others.	2	2
	I felt full of love.	1.86	2.33
	I felt full of loving feelings.	1.71	2.14
Ketamine factor	My motor control felt strangely impaired.	2.57	2.71
	I felt weirdly dissociated.	2.43	2.71
	I felt detached or dissociated from my body.	2	2.57
	I felt detached or dissociated from my emotions.	2	2.29
	My awareness of my body in space felt strangely altered.	1.86	2.71
	I felt like I was floating along in space.	1.86	2.29
Psychedelic factor	I experienced crisp, geometric, flickering patterns.	2.29	2.43
	I experienced insight into the nature of my environment.	2.29	1.86
	I saw dynamic/moving geometric patterns.	2	2.29
	With eyes open, I saw swirling, morphing or flowing illusions.	2	2.29
	I could see especially deeply into my own mind or psyche.	1.71	2.29
	I could see deeply into the human mind.	1.57	2.29

Final items for the PKM scale. Items are sorted into their three respective factors for MDMA (blue, top), ketamine (green, middle) and psilocybin or a classic psychedelic (red, bottom). Items are ranked within their categories by specificity and—when specificity is equal—sensitivity.

### Online survey results

One thousand two hundred and fifty-four survey responses were collected, of which there were 206 fully complete data sets. Participants were required to have used all three drugs before in their lives to be eligible for the survey. Males and females over 18 could complete the survey. We chose not to collect participant demographics. We elected to only compute results for the 206 complete datasets. The following analyses were all applied to these data.

### Factor structure of the *PKM* and its relationship to drug class

The three-factor solution aligned well with our theoretical expectations, with the *PKM scale* item loadings for factors 1–3 picking out prototypical facets of the MDMA, ketamine, and psilocybin/psychedelic experience, respectively (Figure 1). The four-factor solution parsed the psychedelic items further into two subcategories, namely, items pertaining to visual perceptual effects, and items pertaining to insight into the mind/psyche. For clarity of exposition, we present results for the three-factor solution in the main text. However, all findings generalize to the four-factor solution, which is presented in Supplemental Figure S2.

**Figure 1.**
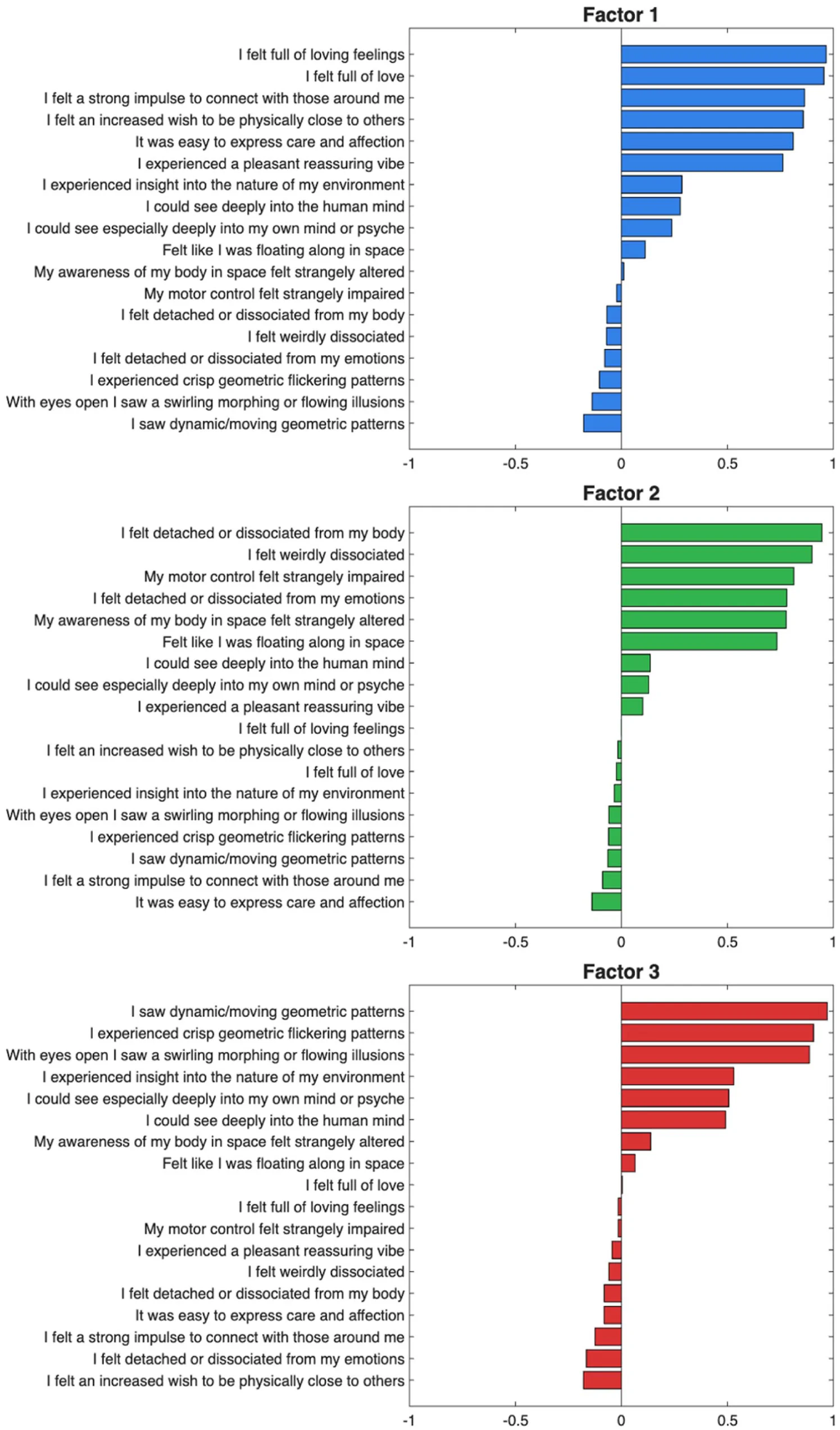
Factor loadings of the final 18-item *PKM* scale according to the 3-factor EFA solution, sorted for each factor independently. Factor 1, in blue, implies MDMA-specific effects (M-factor); factor 2, in green, represents ketamine specific effects (K-factor), and factor 3, in red, represents psilocybin/psychedelic-specific effects (P-factor). Factor loadings for a four-factor solution can be seen in Supplemental Figure S2. EFA: exploratory factor analysis.

To quantify the intuition that the three-factor solution picked out drug-specific phenomenology, we plotted each drug experience rating in low-dimensional factor space. This demonstrated a clear geometric separation of psilocybin/psychedelic, ketamine/dissociative, and MDMA/empathogenic experiences (‘empathogen’ means ‘generating or promoting feelings of empathy’). When the centroid was plotted for each drug category, stratified by dose quartile, this separation pattern became even clearer, with evidence for an increase in the separation as a function of dosage level (i.e., experience intensity), albeit separation was most pronounced in a mid-to-high dose range (Figure 2).

**Figure 2.**
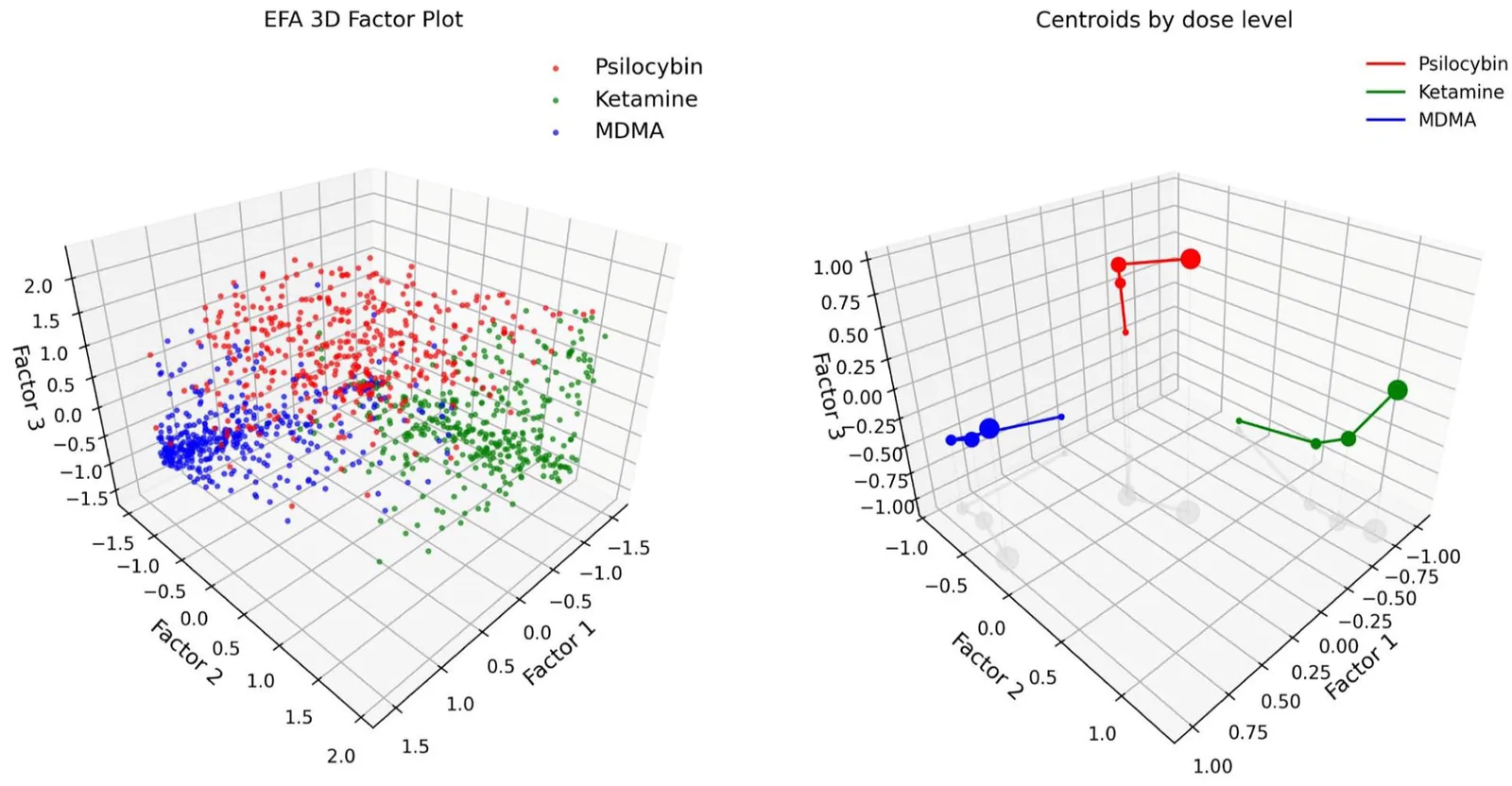
Three-factor model. Left: Each dot represent a participant’s scores, with the drug they were intending to rate shown in one of three colors, red for *psilocybin*, green for *ketamine* and blue for *MDMA*. *MDMA* is most associated with positive loadings on factor 1 (M-factor); *ketamine*, with positive loadings on factor 2 (K-factor); and *psilocybin*, with positive loadings on factor 3 (P-factor). Right: Increasing dosage shown by filled-circle size. *Psilocybin* (red) *Ketamine* (green) move along their respective factors with increasing dose but *ketamine* and *MDMA* begin to load more positively onto factor 3, the P-factor (P for *psilocybin* or *psychedelic*) with extreme high doses. Similarly, *psilocybin* begins to load onto factor 2, the K-factor (K for *ketamine*), at extreme high doses, as does MDMA. This can also be seen in Figure 3.

When plotting each drug’s mean score, stratified by dosage quartile, for each factor separately, we found a striking pattern: namely, as hypothesized, each factor picked out one drug category specifically. Moreover, also as hypothesized, there was evidence of dose-dependency, also referred to as effect *sensitivity* (i.e., increasing factor score as a function of dosage quartile). Interestingly, at the extreme higher dosages there was some evidence that factors began to lose some of their drug specificity. See the ‘Discussion’ section for possible explanations for this.

**Figure 3.**
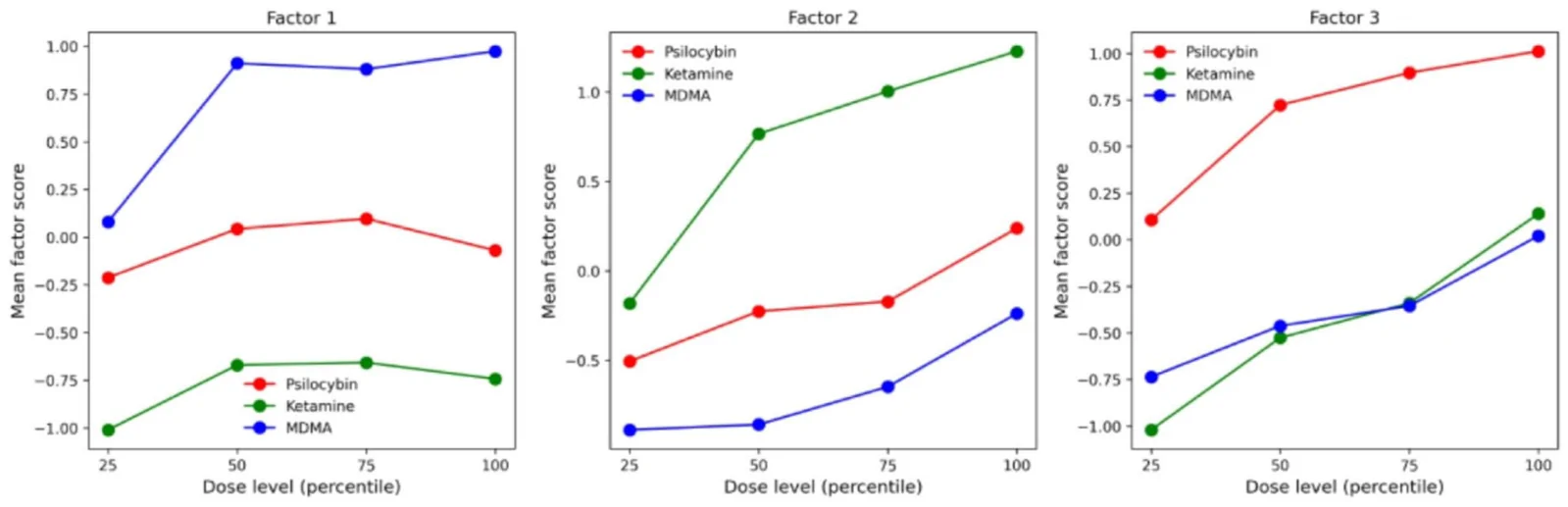
Higher drug doses tend to increase the intensity of each factor, particularly for factors 2 (ketamine/*dissociative*) and 3 (psilocybin/*psychedelic*). Rightmost plot: extreme high doses of *ketamine* (and to a lesser extent, *MDMA*) become more associated with factor 3 (*psychedelic*) effects and even begin to match in intensity the (*psychedelic*) effects of the lowest dose *psilocybin*. Similar dose-dependent effects on factor *incongruity* can be seen for factor 2 (*dissociative*) scores (center plot), where, for example, high dose psilocybin becomes ‘as dissociative’ as lowest dose *ketamine*.

We used supervised classification analysis and geometric centroid analysis to formally quantify the ability of the three-factor solution to discriminate between drug classes. We first trained a supervised multiclass classifier (*k*-nearest neighbors, *k* = 5) to discriminate between drug classes based on the 3D factor scores alone, and tested generalization performance (balanced accuracy) in 10-fold cross-validation. This revealed a high generalization accuracy was 80%, far exceeding the mean accuracy observed under a permutation-derived null model (*p* < 0.001, mean permutation accuracy = 0.33; Figures 4(a) and 5(a)). Next, we quantified the drug × drug centroid separation (Euclidean distance) and compared this to a permutation-derived null distribution. This, too, demonstrated highly significant separation of drug experiences by the three-factor solution (observed mean centroid distance = 1.72, which is significantly greater than the mean distance under the permutation null = 0.11, *p* < 0.001; Figures 4(b) and 5(b)).

**Figure 4.**
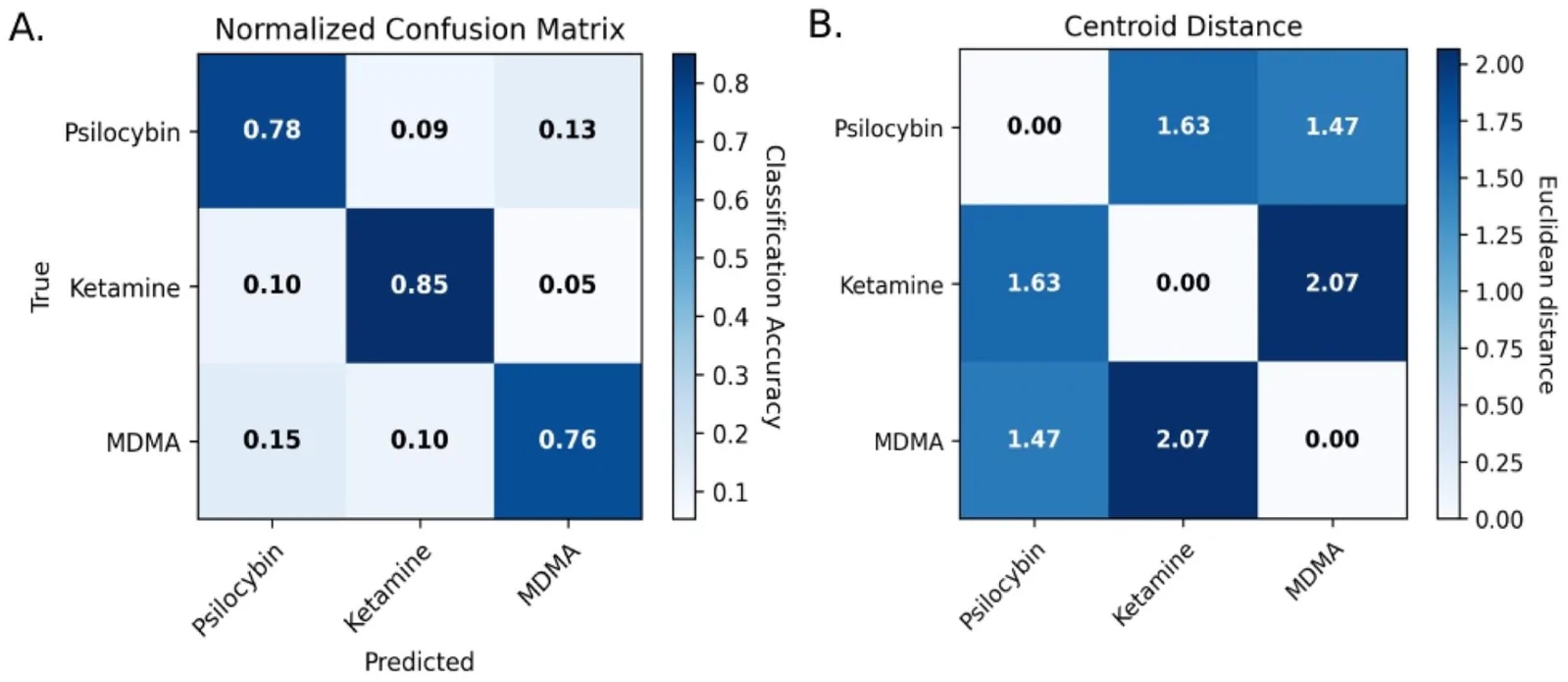
Supervised classification analysis (a) and geometric centroid analysis (b) could formally quantify the ability of the three-factor solution to (blindly) discriminate between drug classes. In other words, participants’ ratings could predict the referred to drug, with a high degree (75–85%) of accuracy, that is, see values within the on-diagonal cells of matrix A.

**Figure 5.**
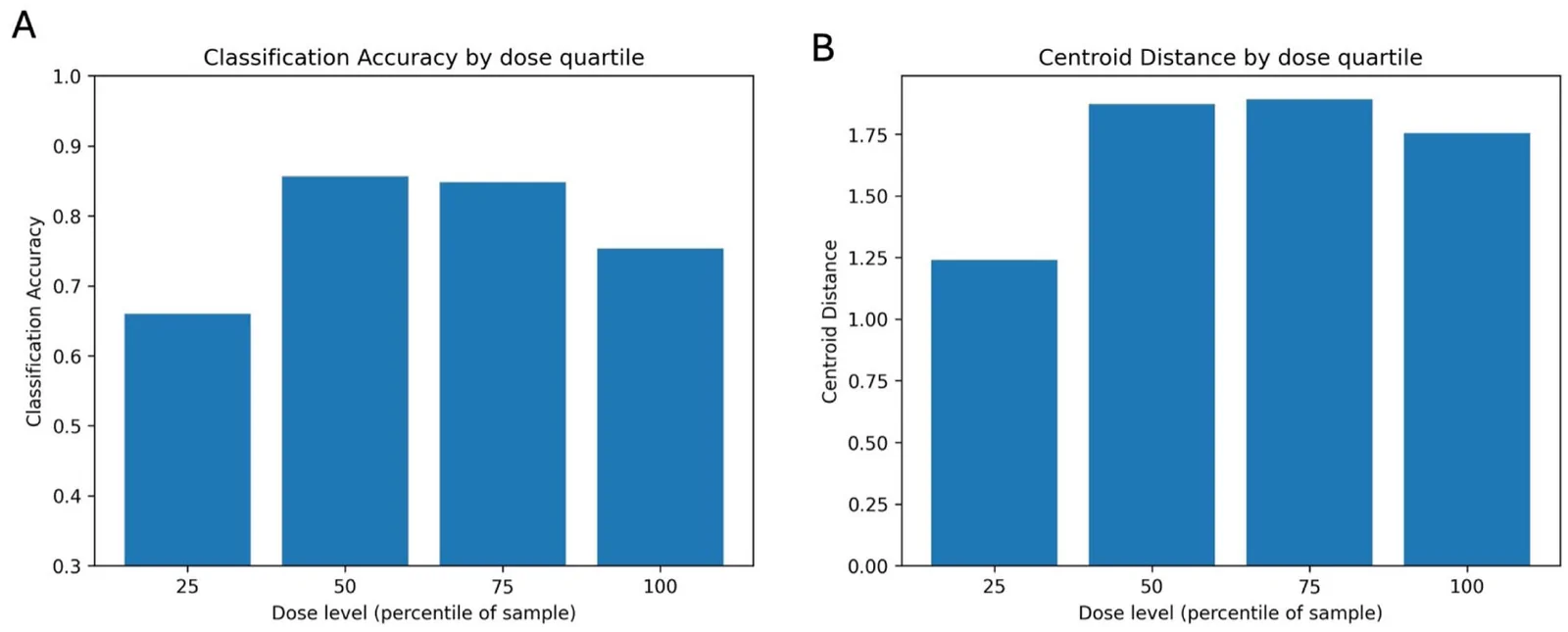
Classifier accuracy (a) and (b) drug versus drug differentiability (by ratings) was maximal within a ‘mid’ or ‘typical’ dosage range and was less impressive at either extreme, that is, the lowest and highest dosages.

Interestingly, both balanced accuracy and centroid separation distance showed an ‘inverted-U’ relationship as a function of drug dosage, confirming the above-mentioned findings that drug experiences become (slightly) less differentiated at the highest reported dosage levels (Figure 5).

## Discussion

Following a novel, mixed-methods approach, this study found a single dimension of subjective experience that could successfully classify psilocybin experiences (a prototypical psychedelic) and differentiate them from other dimensions of experience more closely associated with two other drugs, namely MDMA and ketamine. We call this the *P-factor*, where P can stand for either *psilocybin* or *psychedelic*. Using the P-factor, the correct classification (of psilocybin) could be achieved with greater than 75% accuracy. The P-factor contained items pertaining to both *visual perceptual effects*, such as ‘I saw dynamic/moving geometric patterns,’ and experiences of *psychological insight*, such as ‘I could see deeply into the human mind’. Both factors (*visions* and *insight*) match the etymology of ‘psychedelic’, a term that describes the experience of expanding the content of conscious awareness. A *pro-social feelings* factor and a *dissociation* factor successfully classified MDMA and ketamine, respectively.

To our knowledge, this is the first study to attempt to systematically define ‘psychedelic’ based on phenomenology while simultaneously developing the construct validity of the relevant operational definition. The operational definition we developed here (a six-item P-factor rating scale) can now be tested in future work. Thus, the present work offers the first empirically and etymologically justified definition of ‘psychedelic’.

Future researchers could use the full 18-item *PKM scale* to differentiate between the subjective effects of various psychoactive compounds—or any other induction technique—as a litmus test for determining whether the induced state qualifies as being ‘psychedelic’ or not. To do this, a threshold for P-factor scores could be set (*sensitivity*), and a ratio of P-factor to M and K-factor scores (or some other future developed factor) could also be set to establish *specificity*. Threshold values could be informed by the present work and revised as more data is accrued.

Future researchers might also wish to extend on *the PKM scale* by introducing—and testing—items pertaining to other dimensions of experience, for example, that can be induced by other interventions, pharmacological or otherwise. Some examples include stimulants, sedatives, deliriants—or atypical psychedelics, like ibogaine or 5-MeO-DMT. This approach might help us determine, for example, whether cannabis/THC matches the operational definition of ‘psychedelic’ or elicits a different aspect/dimension of subjective experience as its main action. In this way, we may determine, for example, that THC is a ‘psychoactive cannabinoid’ with some psychedelic-like properties. Future work is now planned to examine ibogaine using similar methods. It is hypothesized that ibogaine will match our operational definition for being a psychedelic.

The 18-item *PKM scale* naturally enquires about (1) *psychedelic-related effects*, (2) *ketamine-related dissociative effects*, or (3) *MDMA-related empathogenic effects*. As above, the validity of our scale and the dimensions of experience it purports to index could be further scrutinized and developed in future work, such as a controlled drug administration study. As part of an iterative process of construct validation, new studies could use the full 18-item PKM or 6-item P-factor scale alongside other more established psychometric rating scales.

The present study has merits but also some significant limitations. We devised a brief and easy-to-implement tool for empirically assaying psychedelic subjective effects (the *PKM scale* and its P-factor specifically), but we do not claim that this scale or factor represents a comprehensive assay for describing and characterizing the varieties of psychedelic experience. It does not do this, and neither was it intended to. Rather, our scale was intended to be a brief tool for capturing quintessential psychedelic effects, differentiable from the main subjective effects induced by similar but different compounds, namely, MDMA and ketamine. There are other methods for classifying whether an inducer is psychedelic or not (e.g., a pharmacology-based classification scheme), and while those have limitations too, they could be combined with a phenomenological assay—like ours— to yield an integrative assay/definition that is more resilient to category errors than any one assay alone.

We acknowledge that the PKM scale and P-factor is too brief to achieve a comprehensive characterization of the psychedelic state. Moreover, even within the P-factor itself, analyses and intuition indicate that a splitting of this factor into two further sub-factors can be justified. Specifically, three of six P-factor items refer to *visual perceptual effects*, whereas the three remaining items refer to more abstract effects pertaining to *psychological insight*. It should also be noted that one of P-factor items, namely ‘I experienced insight into the nature of my environment’ does not overtly refer to *psychological insight*, unless we interpret the effect as being consistent with or relevant to (popular) predictive processing model of perception, that maintains that perceptual processes are (ordinarily) strongly constrained by top-down internal predictive models, also known as ‘priors’. See the ‘REBUS’ model for a relevant account of the impact of psychedelics on mechanisms of predictive processing, and how this might explain—and justify—the inclusion of this specific item in the P-factor (Carhart-Harris and Friston, 2019). In brief, REBUS states that perception becomes unconstrained within the psychedelic state, potentially allowing for more features—of the internal or external environment—to be seen and experienced.

Expanding on a related theme, some might argue that the relevant visual perceptual effects of psychedelics aren’t consistent with the principle that these drugs should be defined by their ability to ‘illuminate latent aspects of the mind’. However, we would naturally challenge this argument. Influential modeling and empirical work on the causal mechanisms underpinning visual perceptual effects, such as the emergence of dynamic geometric patterns—also known as geometric ‘visual illusions’ or ‘hallucinations’, strongly imply (as one might logically surmise) that the effect is caused via the excitation of specific spatiotemporal patterns over the anatomical geometry of the visual cortex itself (Ermentrout and Cowan, 1979; Gulbinaite et al., 2024). The excitation of higher frequency spatial and temporal properties of activity may be a related mechanism underpinning this effect (Atasoy et al., 2017). In a sense, one could logically consider illusory geometric patterns perceived under psychedelics as an example of the principle that these compounds *release*—or make manifest—latent content of the mind, via an excitatory action on brain function.

It is a limitation of the present work that it did not constrain or measure the *context* in which each drug was used. The psychological effects of psychedelics are assumed to be highly context-dependent (Carhart-Harris et al., 2018). and it has been argued, for example, that ketamine has psychedelic—or psychedelic-like—effects when used within a context that is consistent with psychedelic-therapy (Diep et al., 2025). Indeed, the same could be said of MDMA, which, when used in clinical research, is commonly referred to as a ‘psychedelic-assisted therapy’. A natural response to this point is to emphasize that the present work does not deny that MDMA and ketamine have psychedelic effects, but it does invite us to critically question whether quintessential psychedelic effects are the main, defining effect of ketamine and MDMA.

We also note consistent subjective divergences—as those found here—in a pooled analysis of experimental research data from the same research team working with psilocybin, ketamine and MDMA. In this prior work, psilocybin promoted intense visual effects, whereas ketamine promoted intense disembodiment, and MDMA promoted intense states of bliss (Studerus et al., 2010). Poor separation on an ‘insightfulness’ sub-factor was apparent in this prior work, but this sub-factor has poor face-validity for the construct of psychological insight, for example, two of its three items read: ‘I had very original thoughts’ and ‘I felt very profound’.

Another important limitation of the present work relates to its focus on *subjective* methods and data. It is an inherent limitation of phenomenology that words and constructs pertaining to subjective experience are context and culturally sensitive. The language we are operating in is English, and the dominant culture is, broadly speaking, ‘Western’. Within any language and culture, some words are more context and culturally sensitive than others. In the item derivation process, care was taken to avoid highly sensitive and/or framing-dependent terms, as such terms would have negatively impacted the generalizability of our results. Nevertheless, this remains a limitation.

The present work involved an initial Delphi phase that began with the PI proposing a shortlist of subjective items. This process is vulnerable to a priming effect, biasing subsequent item decisions. However, subsequent phases of the Delphi process, namely iteration 3 and 4, involved a democratic procedure, including Delphi members proposing their own items and having them rated by other Delphi members. We acknowledge that Delphi processes have evolved in recent years, and our adherence to guidelines for best practice could be critiqued (Anthony, 2025).

The PI’s collaborative network has done much to develop the construct validity of *psychological insight*, and its relevance to the psychedelic experience (Peill et al., 2022) and the mechanism of action of psychedelic-therapy (Lyons et al., 2024). It should be acknowledged though that other independent, contemporary clinical researchers have also highlighted a key role for psychological insight in the action of psychedelics (Acevedo et al., 2025; Davis et al., 2021; Garcia-Romeu et al., 2020; Kugel et al., 2025), but others have also spotlighted other dimensions of psychedelic experience, such as the so-called ‘mystical-type experience’ (Griffiths et al., 2006), while others have questioned whether putative ‘insights’ are always necessarily lucid, well-reasoned and founded (McGovern et al., 2024)—whatever the induction technique for eliciting them.

In brief, subjective methods lend themselves to framing, priming and perspectival biases (Taves, 2020), where, for example, researchers feel impelled—implicitly (or otherwise)—to perceive and demonstrate the legitimacy of their preferred psychological or philosophical perspective. This said, it was not a contrivance that the P-factor items matched—and therefore validated—the etymology of the term ‘psychedelic’ itself. In this regard, we note the consistency of the present study’s result with a prior finding that experienced drug users consider LSD and psilocybin exceptional tools for eliciting the release of latent or ‘unconscious’ psychological material (Carhart-Harris and Nutt, 2010). In this previous work, experienced drug users regarded only LSD and psilocybin—both classic psychedelics—as compounds ‘definitely’ capable of allowing access to ‘the unconscious mind’. This property was not endorsed with the same conviction for the control compounds: cannabis, MDMA, ketamine and alcohol. Indeed, it was emphatically rejected for alcohol (Carhart-Harris and Nutt, 2010).

The idea that psychedelics facilitate access to latent psychological material invokes obvious associations with principles of psychodynamic psychology. Moreover, the etymology of the term ‘psychedelic’—combined with the outcomes of the present work—does lend support to the view that *psyche-illumination* is the principal, defining action of psychedelics. Some may interpret this as an exciting development for—and potential validation of—principles of psychodynamic psychology (Grof, 1979), whereas others may wish to deepen their critique of the present study’s methodology, findings, and implications, perhaps because of an opposition to the legitimacy of psychodynamic psychology (Chater, 2018). Any such critique should be welcomed, if well-reasoned and empirically supported. Equally, however, fresh empirical efforts to test and potentially support the hypothesis that psychedelics principally work to *elicit the release of latent psychological content* should be welcomed. If it is established that this is indeed what psychedelics principally do, then it could—and perhaps *should*—have far-reaching implications.

Returning to the inherent limitation of subjective measures and reasoning, the present work was guided by a *dual-aspect monist* philosophical perspective, which gives equal weight to the validity of human subjective experience as it does to its encoding biology. From a cognitive neuroscientific perspective, the quest is to find a sensitive and specific functional code (a biomarker) that bridges between the two relevant domains, phenomenology and biology. We have taken the position that initiating pharmacological actions (e.g., serotonin 2A receptor signaling) is an insufficiently reliable biomarker. In time, however, we suspect that better biomarkers will be found. Even if we were to have a perfectly reliable mapping, this would remain a map (biology) to territory (subjective experience) relationship (Frank et al., 2024). In this sense, the approach known as ‘neurophenomenology’ is relevant (Timmermann et al., 2023).

Phenomenology, that is, human subjective experience, is treated as primary here because of its direct relevance to the etymology of ‘psychedelic’—and its fundamental validity and importance more generally. However, we acknowledge the importance and potential power of identifying a good biological counterpart to the relevant subjective phenomena—akin to the value of a good map. If we can find, for example, a specific spatiotemporal pattern of brain activity that reliably maps to a relevant subjective phenomenon, this will enrich our account of—and ability to induce and predict—the latter. Through this approach, we may be able to overcome some of the inherent limitations of subjective methodologies, such as perspectival-bias related quibbles over constructs, epistemic frameworks, and their respective validities. For example, questions such as: *is psychological insight*, *ego-dissolution or mystical experience the core of the psychedelic experience?*—will appear somewhat redundant if—and when—we come to know how the relevant experiences are biologically encoded. For example, if it is found that a particular quality of brain function encodes all three types of experience, then this would imply that the experiences rest on a largely consistent biology and are, therefore, largely consistent constructs. More briefly, it would imply that they refer to the *same essential thing*, in mind and/or brain, that only differ in relation to subjective framing biases.

One interesting observation of this study was that, at the extreme high dosages, there was some evidence that factors began to lose their drug-to-factor *specificity* or *congruity*, for example, at extreme high doses, ketamine and MDMA begin to behave more like a classic psychedelic; note, we found this effect for the four-factor EFA solution also, across both ‘visual perceptual’, and ‘mind-revealing’ psychedelic factors. This finding may reflect non-specific biological actions at higher doses (e.g., non-specific receptor binding and agonist and neurophysiology that follows). For example, 5-HT2A receptors would become more saturated by higher concentrations of serotonin with high dose MDMA, causing it to act more like a direct 5-HT2AR agonist classic psychedelic (Brouwer and Carhart-Harris, 2020), and high dose ketamine could also cause some 5-HT2AR agonism and/or asynchronous glutamate release and cortical desynchrony—mirroring a downstream action of classic psychedelics—the so-called ‘entropic brain effect’ (Carhart-Harris, 2018; Carhart-Harris et al., 2014; Peill et al., 2022; Schartner et al., 2017; Siegel et al., 2024).

The loss of drug-to-factor congruity at higher doses may also be tied to other factors that separate low from high dose drug reports, such as the duration of time elapsed since experience (higher-dose experiences lasting longer) or participant-level subjective reporting biases, for example, some individuals may have felt inclined to inflate their ratings for the highest doses taken, for example, to emphasize the generic intensity and novelty of the relevant altered state of consciousness. These results may also, however, be a mere statistical phenomenon, occurring due to a ceiling effect for within-factor, drug-congruent scores; meaning that, at extremely high doses, drug-congruent scores ‘max out’, while drug-incongruent scores still have freedom to increase.

In conclusion, the present work sought to examine the construct validity of an operational definition of ‘psychedelic’ based on a six-item P-factor subjective rating scale. This factor classifies an inducer as ‘psychedelic’ if it induces item-described *visions* and *psychological insight*. Our findings lend support to the position that the classic psychedelic drug, psilocybin, elicits these subjective effects as its main action, while other (comparator) compounds, MDMA and ketamine, elicited pro-social feelings and dissociative effects (respectively) as their main actions. The present work represents a positive contribution to the ongoing debate over how to define ‘psychedelic’, underlining the need to sharpen our definitions while proposing how we might begin to do so.

## Supplemental Material

sj-docx-1-jop-10.1177_02698811261464986 – Supplemental material for Defining ‘psychedelic’ Supplemental material, sj-docx-1-jop-10.1177_02698811261464986 for Defining ‘psychedelic’ by A. E. Ostrand, M. M. Nour, C. Timmermann, A. Rosati, L. X. Luan, A. Gomez-Emilsson, J. Bornemann, K. T. Greenway, L. Roseman and Carhart-Harris R in Journal of Psychopharmacology
