# Supplementary material for: Defining ‘psychedelic’
Source: J Psychopharmacol. 2026 Aug 17;40(11):1760–70. doi: 10.1177/02698811261464986 (PMC13743727; doi:10.1177/02698811261464986)
Supplement: Supplementary file 1 [file sj-docx-1-jop-10.1177_02698811261464986.docx]

Defining psychedelic

Supplemental Material

**Figure S1A:** First pass Items of the Delphi in order of specificity scores. Where it is written e.g., “1xP”, this means that 1 person within the Delphi chose Psilocybin for an item intended to describe the effects of a different drug. Any such cases negatively impacted the specificity score.

**Figure S1B:** Second pass items of the Delphi in order of specificity scores.

**Figure S1C: Third pass items of the Delphi in order of specificity scores.**

**Figure S1D: Fourth pass items of the Delphi in order of specificity scores.**

**Figure S2.** Factor loadings of the 18 *PKM* items according to the 4-factor EFA solution, sorted for each factor independently. Factor 1, in blue, implies MDMA-specific effects; factor 2, in green, ketamine specific effects, and factors 3 and 4 (in red and dark red, respectively) imply psilocybin/psychedelic specific effects, namely, factor 3 implying *visuals* and 4, *psychological insight*.

**Table S1. Preset list of dosages in ascending order for 3 drugs (Psilocybin, Ketamine, MDMA).** Participants were asked to select both a typical dose and the dose that produced the most intense effect they have ever experienced for all three drugs. The preset list of dosages is presented in the table.

**Figure S3.** Supervised classification analysis (A) and geometric centroid analysis (B) of the 4-factor solution.

**Figure S4.** Classifier accuracy (A) and (B) drug vs. drug differentiability (by ratings) for the 4-factor solution.

**Figure S5**. Dose Quartiles for each Psilocybin, Ketamine and MDMA.

**Figure S6.** 1D Mean Factor Score by Dose for the 4-factor solution. Factor 1 describes MDMA-like effects, factor 2 represents dissociative (ketamine) specific effects, and factors 3 and 4 represents Psychedelic-like effects; 3 being more related to visions and 4 being more related to psychological insight.

**DELPHI**

**DELPHI METHODS**

The Delphi was comprised of eight individuals, four males (CT, LR, KG, RCH) and four females (JB, LL, AO, AR). Delphi members were chosen by the principal investigator (RCH) based on his knowledge of these individuals and assessment that they have a sufficient depth of knowledge regarding the phenomenology of all three relevant compounds. The Delphi included a range of professional researchers studying psychedelics and individuals with prior experience delivering psychedelic-therapy. Delphi members were asked not to confer with each other. Email correspondences were done 1:1 with the PI rather than always as an entire group. This approach helped mitigate the risk of social conformity or alignment biases. There were three phases to—or iterations of—the Delphi process. Descriptions of these can be found in the supplementary material.

**First iteration**:

The first iteration was led by the PI (RCH). Eighteen candidate subjective rating scale items were proposed, by RCH, to all members of the Delphi. Based on clinical research experience involving all three of the focal drugs, namely, psilocybin, MDMA, and ketamine, the PI created items felt to well represent the subjective effects of each. Six items were created for each drug; thus, 18 items were created in total.

(Step 1) Delphi members were briefed that— for each of the 18 items—they should select just one of the three focal drugs, namely, psilocybin, MDMA, or ketamine, according to their perception of which drug most reliably produces the effect described by a given item.

Next, (Step 2) they should rate each item—that they ascribed to just one drug (in Step 1)— on a 0-3 scale (0 = not at all specific; 3 = entirely specific)—according to how *specific* they feel it is for their chosen drug versus the two non-selected alternatives. If members felt that the item described effects that were just as much a feature of another (non-selected) compound, then they were instructed to ascribe the lowest possible rating, i.e., 0 (= not at all specific); however, if they felt that the effect was entirely specific or exclusive to the selected drug, they gave it a rating of 3 (= entirely specific).

Delphi members were also asked to rate ‘sensitivity’, which we defined as how easily the chosen drug could product the described effect. We concretized this as the dose-dependent ease or reliability with which the drug could produce the described effect. *Sensitivity* was also rated on a 0-3 scale, where a score of 3 implied that higher doses of the relevant drug reliably induce the described effect, whereas a score of 0 would suggest that— no matter the dose—the effect couldn’t reliably be produced. In this way, each Delphi member rated each item, and the drug that they ascribed to it, according to two dimensions: the *specificity* and *sensitivity* with which that chosen drug could produce the described effect.

**Second iteration:**

In Step 1, Iteration 1, one of Delphi members selected one (preferred) compound (*ketamine*) multiple times, which was a non-standard strategy when compared with other members of the Delphi. It was suspected that this occurred due to the relevant individual having a disproportionate depth of prior research and/or clinical experience with a particular compound (ketamine) relative to the others. Thus, in a second iteration of the Delphi process—Step 1 was repeated, with the same 18 items, albeit this time with the instruction that members should select an equal number of items for each type of drug, i.e., they had to choose 6 items for psilocybin, 6 items for ketamine, and 6 items for MDMA. This was Iteration 2.

**Third iteration**

For the third iteration, Delphi members were invited to create their own items. They were instructed that each item should describe subjective effects that they feel are specific to one of the three focal drugs. Each member was asked to create 3 items in total, 1 for each of the 3 focal drugs. This produced a fresh list of 24 new items. The PI supplemented this list with 5 more items (and subtracted 2) based on the results of iterations 1 and 2 and deeper reflection on drug-specific effects of each focal compound. This created a total list of 27 items. These 27 items were subsequently anonymized, randomized, and sent back to Delphi members to be rated as per Step 1 and 2. To avoid confirmation bias, Delphi members were not allowed to rate their own suggested items. If they ignored this instruction or failed to follow it, we knew not to score the relevant items when computing the results.

**Fourth iteration**

In a fourth and final iteration, the PI consulted the co-founder of a non-profit organization (AEG), the Qualia Research Institute, focused on efforts to better understand and characterize exceptional subjective experiences, such as those produced by psychedelic and other novel psychoactive drugs plus other induction methods. This individual was briefed on the aims and methodology of the study and invited to create some new items that he felt were specific to each of the three focal compounds. Two items from the third iteration were revised and included in this fourth iteration based on 1) their good specificity scores in the third iteration and also 2) a desire by the PI that the items be succinct. The resulting 10 items were rated by the entirety of the original Delphi once more, again using the methodology described for Step 1 and 2 of iteration 1.

Results of each iteration were analyzed by calculating the average *specificity* and *sensitivity* scores for each item. Results for each iteration can be found in the supplement and the final 18 items of the PKM scale are shown in Table 1 of the results section. To comprise the final PKM scale, for each of the three focal drugs, six items each were selected based on the highest *specificity* scores from each and all of the iterations described above. Thus, some items were selected after the results of the second iteration because their good *specificity* had been established already at that point. If there was ambiguity regarding which items to select, *sensitivity* scores were consulted to guide the decision. More formally, items with equivalent *specificity* scores were decided between based on which had the highest *sensitivity* score. This was our methodology for creating and selecting between items felt— and found— to describe effects with a good degree of specificity for each of three focal compounds, psilocybin (P), ketamine (K) and MDMA (M). The final 18-item PKM scale can be viewed in Table 1.

In this way, a new rating scale, *the* *PKM scale*, was produced containing 18 items in total that could be said contain items specific to psilocybin, ketamine and MDMA. We call this ‘the PKM scale’ (‘P’ for psilocybin or psychedelic, ‘K’ for ketamine, and ‘M’ for MDMA). Internally, we pronounced the scale in a way that is phonetically consistent with ‘Pokémon’—the children’s card game, but ‘P K M’ also works well. Next, we sought to test and potentially validate this PKM scale as a tool to discriminate between the subjective effects of the three focal drugs. We did this by consulting a large sample of individuals with self-declared prior personal experience with each compound. We did this using an online survey approach, described below.
